# Knowledge, Attitudes, and Practices Regarding Tick-Borne Diseases in a Non-Lyme-Endemic County in Southeastern Ohio: Results from a Cross-Sectional Survey

**DOI:** 10.3390/healthcare13161940

**Published:** 2025-08-08

**Authors:** Benjamin R. Bates, Cora G. Farra

**Affiliations:** 1 School of Communication Studies, Ohio University, Athens, OH 45701, USA; 2 Infectious and Tropical Disease Institute, Ohio University, Athens, OH 45701, USA; 3 Department of Sociology and Anthropology, Ohio University, Athens, OH 45701, USA; 4 Department of Communication Studies, University of Nebraska—Lincoln, Lincoln, NE 68588, USA

**Keywords:** tick-borne diseases, knowledge, attitudes, practices model, cross-sectional studies, health communication, behavioral practices

## Abstract

Background/Objectives: Tick-borne diseases (TBDs) are a significant public health problem and are expanding to formerly naive areas of the United States, such as the lower Midwest. To counter TBDs, many researchers apply the Knowledge, Attitudes, Practices (KAP) model to identify human-level factors that can be activated in campaigns to prevent tick-bites. These studies are, however, almost exclusively conducted in Lyme disease endemic areas of the US. We sought to outline KAPs among residents of a naïve County in southeast Ohio to provide baseline data to inform future educational and communication campaigns. Methods: To identify KAPs before Lyme disease and other TBDs become endemic, we conducted a cross-sectional survey to collect data on exposure to ticks and to examine KAPs regarding TBDs in a non-Lyme-endemic county in Ohio that borders a Lyme-endemic county in West Virginia. Results: Two hundred thirty-one people completed the survey. Most participants reported time spent in places where ticks are common and about half reported having a tick on themselves, but low levels of concern that they would be bitten or contract a TBD. Participants reported high levels of awareness of Lyme disease but low levels of awareness of other TBDs. The perceived seriousness of TBDs was low. Participants reported suboptimal adoption of all TBD-prevention behaviors. The most common barriers were forgetting to perform a preventive practice or not being aware of a preventive practice. Conclusions: These gaps in KAPs provide clear targets for public health communication messages to prevent tick-bites, particularly in non-Lyme-endemic counties in the US.

## 1. Introduction

Tick-borne diseases (TBDs), such as Lyme disease, Rocky Mountain spotted fever, and Powassan virus disease, are a significant public health problem in the United States [1,2,3]. These diseases, and the ticks that spread them, are most commonly found in the Upper Midwest and New England states. As humans increasingly live in the wildland-urban interface [4,5] and as climate change warms previously low-risk areas [6,7], the presence of ticks in naïve areas is growing, raising the threat of TBDs in formerly resistant areas. Although our understanding of the biology and genetics of ticks and TBDs has grown rapidly in light of these challenges, public health and health communication efforts have not kept pace. In 2008, Piesman and Eisen argued, “success stories in the fight against tick-borne diseases are lacking. Both new approaches to tick and pathogen control and novel ways of translating research findings into practical control measures are needed to prevent tick-borne diseases in the twenty-first century” [8]. Compared with other diseases, the study of Knowledge, Attitudes, and Practices (KAPs) associated with TBDs is relatively new [9]. Herrington’s pioneering study established that fewer than half of people regularly adopt tick-bite prevention behaviors, and fewer than half believed themselves to be at risk of Lyme Disease, but that those who believed themselves to be at risk were eight times more likely to enact preventive behaviors [10].

Since Herrington’s study, and since Piesman and Eisen’s call to action, researchers have begun employing KAP questionnaires to determine the communication and information needs of populations experiencing different levels of risk from tick-borne pathogens. KAP questionnaires are particularly important in infectious disease contexts as choosing to adopt or not adopt preventive measures is guided by knowledge of the disease, attitudes toward the disease and toward the preventive practice, and intentions to adopt recommended practices [11,12,13,14,15]. In addition to explaining behavior, assessing KAPs is useful for informing the design of public messaging about tick control and tick-bite prevention behaviors [1,2,16,17].

Generally, knowledge levels regarding TBDs are inadequate and the links among KAPs are unclear. Most KAP studies have assessed health-related professionals or populations particularly likely to be exposed to ticks. Among various professional populations, there are low levels of knowledge of ticks and TBDs, and limited relationships between knowledge, attitudes, and tick-bite prevention practices, including work among physicians [18,19,20,21], health department employees [22,23], health professionals working in schools [24], and school administrators [25]. Indeed, most studies found that professionals inadequately understood treatment and prevention practices. Across these professional populations, researchers have called for increasing the amount of knowledge as a means of promoting attitudes favoring tick-bite prevention practices, with the goal of adopting these practices in the populations these health professionals serve. Similarly, persons working in higher risk occupations, such as farmers [26], forest management workers [27,28], and park rangers [29], display low to moderate levels of knowledge and dismissive attitudes toward risk that may interfere with the uptake of preventive practices. The same pattern has been found among equine caretakers [30], dog owners [31], and cat owners [32], all of whom are more likely to encounter ticks while caring for their animals.

General population surveys in places where ticks are endemic show that there are low to moderate levels of public knowledge related to ticks and that risk attitudes are predictive of intentions to adopt tick control practices. Much of the focus has been on Lyme-endemic places such as New England [17,33], the Mid-Atlantic states [3,16,34,35,36,37,38], and the Upper Midwest [2,39]. Yet, studying low-incidence states is necessary. Bayles and his colleagues engaged people living near St. Louis, Missouri, despite Missouri having low endemicity of Lyme disease [1]. Because the risk behaviors for Lyme disease and Rocky Mountain spotted fever (which is endemic in Missouri) are the same, studying KAPs in low-Lyme incident states may provide insight into broader patterns of tick-born disease prevention and protect people in Missouri when Lyme-positive ticks begin to enter the state.

Despite this need to address non-Lyme-endemic states, very limited research has been conducted in the lower Midwest, with Omodior and colleagues’ studies of adults and of parents in Indiana [40,41] and Chakraborty et al.’s study of farmers in Illinois [26] being the examples. The lower Midwest comprises mostly low Lyme-incidence states, and KAP studies of tick-borne disease are rarely carried out there. This may be because people living in high-incidence states are in greater need of messaging [41]. However, they also argue that low-incidence states neighboring a high-incidence state should also receive messaging. It is curious, then, that the state of Ohio has not been made subject of a KAP questionnaire related to TBDs. Ohio borders two Lyme-endemic states (Pennsylvania and West Virginia) as well as bordering two low-incidence states where KAP questionnaires have been applied (Michigan and Indiana). Moreover, Ohio is populated by American dog ticks, black-legged ticks, and lone star ticks, which can carry a variety of TBDs [42], and the numbers of these ticks are increasing as average ambient temperatures rise statewide [43].

As these ticks spread to Ohio, and infection with TBDs becomes more likely, understanding KAPs in Ohio related to TBDs becomes more important if we wish to promote preventive practices. Given this need to communicate tick prevention behaviors more effectively into a naïve population and given that establishing a current baseline for KAPs will aid in assessing the effectiveness of communication efforts in the future, we ask what the current state of KAPs is in a naïve county in Ohio. The objective, therefore, is to outline KAPs among residents of a naïve County in southeast Ohio to provide baseline data to inform future educational and communication campaigns.

## 2. Materials and Methods

### 2.1. Ethical Considerations

The study was conducted according to the guidelines of the Declaration of Helsinki, and approved by the Institutional Review Board of Ohio University (protocol code IRB-FY25-218, 5 November 2024). All participants in the study had reached the age of majority and provided written informed consent.

### 2.2. Study Design

To outline current KAPs, a cross-sectional, non-probability sample design was employed. The questionnaire was distributed in a low-Lyme-endemic county in Ohio (Athens County) that borders a high-Lyme-endemic county in West Virginia (Wood County). Athens County was selected because of this border status. Border counties share similar habitats for ticks and similar socio-demographics for humans but have different encounters with TBDs themselves [44]. Examining the low-endemic county allows us to understand KAPs among a naïve population and to intervene before confounds from tick-disease exposure emerge.

The questionnaire used the 2019 Upper Midwest Tick-borne Disease Prevention Survey [2], with minor modifications for use in Ohio (e.g., county names). This questionnaire comprises a mixture of binary, multiple response, and Likert-type (1–5) measures. It has five sections: section one assesses knowledge of TBDs and risk related to tick bites; section two assesses current use of tick prevention behaviors and associated barriers; section three assesses willingness to perform personal and environmental tick prevention behaviors; section four assesses communication channel preferences; and, finally, section five assesses demographics. The study protocol was reviewed and approved by an Institutional Review Board (IRB-FY25-218).

### 2.3. Data Collection

The questionnaire was distributed through intercept methods in the winter of 2025. Winter was chosen because ticks are generally not active in southeast Ohio during these months and messages from the Ohio Department of Natural Resources and from the Ohio Department of Health about ticks are generally distributed in summer.

**Figure 1:**
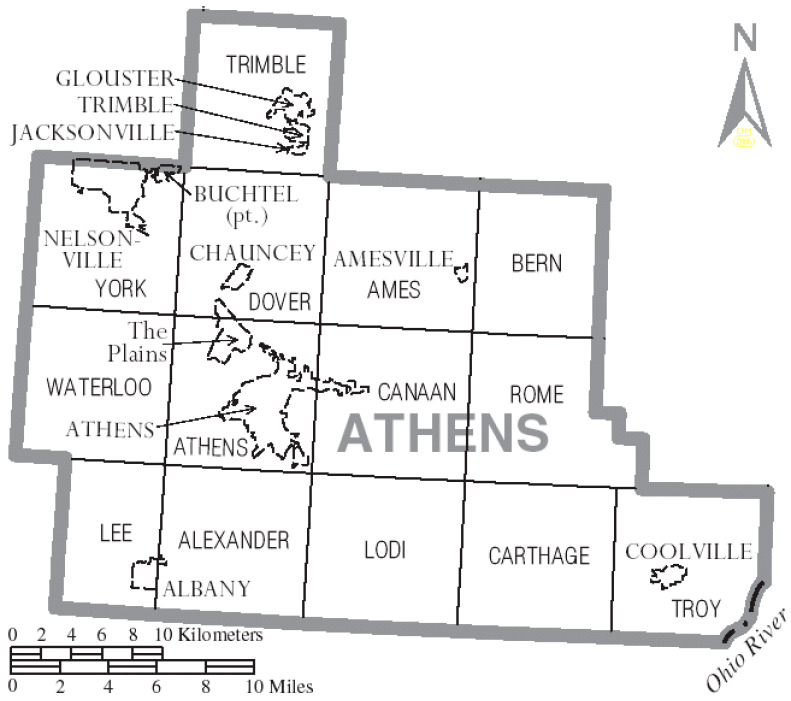
Map of Athens county, Ohio with municipal and township labels, reproduced from wikimedia commons under an attribution-sharealike 3.0 unported license (https://commons.wikimedia.org/wiki/File:Map_of_Athens_County_Ohio_With_Municipal_and_Township_Labels.PNG), accessed on 23 July 2025.

Sixteen undergraduate students were trained in human subjects research protections and, as part of their undergraduate class in Health Communication, collected questionnaire data. Participants were intercepted in public places in the towns/cities of Albany, Athens, Nelsonville, Coolville, and the Plains or were met at their homes in the surrounding County (See Figure 1). Participants were required to be at least 18 years of age and be residents of Athens County. Participants were asked to read and sign an informed consent form and to then complete pen-and-paper surveys of approximately 10 min of duration. The questionnaire was in English only. Participants did not receive compensation for their participation.

### 2.4. Data Analysis

Following Beck and her colleagues’ analysis of the 2019 Upper Midwest Tick-borne Disease Prevention Survey [2], we began our analysis of the data with simple descriptive statistics. In the 2019 study, Beck et al. conducted a series of *t*-tests and Chi-square analyses, and concluded with a regression analysis; we sought to do the same. G*Power 3.19.7 was used to determine the minimum sample size. For the *t*-tests, using standard assumptions of a predicted effect size (Cohen’s *d*) = 0.5, and with α = 0.05 and β = 0.80, a minimum of 176 participants was needed. For the Chi-square analyses, using standard assumptions of a predicted effect size (Cramer’s *v*) = 0.3, and with α = 0.05 and β = 0.95 where the largest degree of freedom = 2, a minimum of 172 participants was needed. Finally, for a regression model with 32 candidate predictor variables, using standard assumptions of a predicted effect size (f^2^) = 0.15, and with α = 0.05 and β = 0.95, a minimum of 267 participants was needed. For all analyses, statistical significance was set at *p* < 0.05 and Cohen’s cut-offs for interpreting effect size were used. All data were analyzed with SPSS 28.0.

## 3. Results

The questionnaire was distributed in Athens County, Ohio, and a total of 231 participants submitted responses to the survey. One questionnaire was excluded because the person completing it was not a resident of Athens County, leaving 230 completed survey submissions included in the analyses. Because only 230 participants were successfully recruited we did not have sufficient participants to run the regression analysis, but we were able to meet the requirements for the *t*-tests and Chi-Square analyses.

### 3.1. Participants

The final sample was fairly representative of Athens County. Participants included 142 women (61.7%), 85 men (37.0%), and 3 people who identified as something else (1.3%). Participants ranged from 18 to 81 years of age (M = 22.3, s.d. = 7.5). Participants were mostly non-Hispanic (215, 93.5%) and white (215, 93.5%). Participants reported self-defined type of residence; the most common type was in a town (116, 50.4%), followed by suburban residency (64, 27.8%). The majority had received some education at a college (68.3%). The most common level of income was less than $25,000 per annum (107, 46.5%). Compared to census estimates for the County surveyed, the participants were younger and had generally higher levels of education, likely reflecting the presence of a large university in the County. See Table 1 for complete demographics.

### 3.2. Knowledge of Ticks and Tick-Related Disease

Most participants spent at least some time in places where ticks were present. Eighty-five participants (37.0%) spent time weekly in places where they perceived ticks to be present between the months of April through October, and 67 spent time in these places at least once a month (29.1%). Less common were daily time spent in these places (18, 7.8%), time spent less than once or twice a month (35, 15.2%), and time spent only one or two times from April through October (21, 9.1%). Of 226 participants who spent any time between April through October in places with ticks (98.3% of the sample), participants reported that they were unlikely to encounter ticks around their homes (M = 1.88, s.d. = 1.10), around a cabin or vacation home (M = 1.57, s.d. = 1.54) or while at work (M = 1.31, s.d. = 0.93). The participants reported it was somewhat likely they would encounter ticks at recreational areas in the community (M = 2.65, s.d. = 1.12) or in other locations (M = 2.47, s.d. = 1.13). In no place did the expectation that one would encounter ticks exceed the midpoint on these 5-point scales. A slight majority of all participants reported having never removed a tick from their skin or clothing at least once in their life (122, 53.0%), but only 36 of the participants had done so in the last year (15.7%).

Almost all participants had heard of Lyme disease (220, 95.7%), and perceived Lyme disease as very serious (M = 4.05, s.d. = 1.16). As shown in Table 2, awareness of Rocky Mountain spotted fever, anaplasmosis, babesiosis, ehrlichiosis, and Powassan virus disease was lower, and these TBDs were seen as less serious. Overall, participants perceived TBDs as fairly uncommon in their community (M = 2.47, s.d. = 1.01). When participants were asked how likely it was that they or another person in their household would get a tick-borne disease in the coming year, they indicated on average that was unlikely (M = 1.68, s.d. = 0.80). Overall, only 19 participants reported that they or a member of their household had been diagnosed with a tick-borne illness (6.1%). Among these, five participants reported a diagnosis in the past year (2.2%), six in the previous one to five years (2.6%), and eight more than five years ago (3.4%).

### 3.3. Tick-Bite Prevention Behaviors and Reported Barriers

Two hundred and twenty-six participants reported that they spent at least some time in places where ticks could be present from April through October. Of these individuals, about one-quarter, 55 (24.3%), reported that they check themselves thoroughly for ticks most of the time and 17 (7.5%) reported they check themselves every time. Participants who selected anything other than “always” checking were asked to endorse up to five pre-defined barriers that prevented them from checking for ticks as well as being able to name an additional barrier. Among the 209 individuals who did not report *always* checking for ticks thoroughly, the most commonly reported barrier was forgetting to check (172, 82.3%). Only about one-third of participants endorsed each of the other barriers of thinking it too much trouble (49, 23.4%), not thinking they would find a tick (55, 26.3%), checking but not checking thoroughly (60, 28.7%), or not being worried about ticks (72, 34.4%). As indicated in Table 3, people who were more frequently in places where ticks are common, and people who believed their community and/or household was at risk of TBDs, were more likely to check themselves for ticks. These differences were statistically significant and the effect size for each was moderate (*d* = 0.60, *d* = 0.55, *d* = 0.47, respectively).

Among these participants, 52 (23.0%) reported that they use bug repellent most of the time and 12 (5.3%) reported they use bug repellent every time. Among the 213 individuals who did not report *always* using bug repellent, the pattern of barriers was the same. Participants who selected anything other than “always” checking were asked to endorse up to six pre-defined barriers to using repellent as well as being able to name an additional barrier. The most common barrier was forgetting to apply repellent (170, 79.8%). The barriers of being concerned about the cost of repellent (26, 12.2%) or the safety of repellent (17 (8.0%) were relatively rare, and other barriers of not knowing that repellent was effective (50, 23.5%), not liking repellent (76, 35.7%), or not being worried about ticks (55, 25.8%) were less common. As indicated in Table 3, individuals who believed their community was at risk of TBDs were more likely to use repellent. This difference was statistically significant and the effect size was moderate (*d* = 0.48).

Among all participants, 36 (15.7%) reported that they had ever treated their home, cabin, or vacation home with a pesticide to kill ticks. Participants who selected not treating or not knowing if they had treated their home were asked to endorse up to six pre-defined barriers to home treatment as well as being able to name an additional barrier. Most barriers to treating the home with pesticide were heavily endorsed by these participants, including no knowing that pesticides were available (195, 84.8%), that applying them seemed like too much work (188, 81.7%), concerns about cost (189, 82.2%) and healthiness (201, 87.4%) of application, and a lack of worry about ticks on the property (169, 73.5%). The only barrier not endorsed by a majority of participants was not owning the property, but still 107 (46.5%) endorsed this barrier. As indicated in Table 3, people who believed their community and/or their household to be at risk of TBDs were more likely to have used these pesticides. These differences were statistically significant and the effect size for each was moderate (*d* = 0.58, *d* = 0.64, respectively).

Among all participants, only 10 (3.9%) reported that they had ever treated their home, cabin, or vacation home with a specialized device that applies pesticides to rodents to kill ticks. Participants who selected not treating or not knowing if they had used a device were asked to endorse up to six pre-defined barriers to use of a device as well as being able to name an additional barrier. Similar to pesticide application, most barriers were heavily endorsed, including not knowing about devices (157, 68.3%), installing them seemed like too much work (196 (85.2%), concerns about cost (202, 87.8%) and healthiness (211, 91.7%) of the devices, not being worried about ticks (183, 79.6%), and not owning the property (181, 78.7%). As indicated in Table 3, people who perceived their household to be at risk of TBDs were more likely to have used these devices. This difference was statistically significant, and the effect size was extremely large (*d* = 1.00).

### 3.4. Willingness to Practice Tick Prevention Behaviors

Participants generally reported willingness to perform tick bite prevention behaviors. About two-thirds of participants (158, 68.7%) were willing to perform tick checks at least once a day (158, 68.7%). About three-quarters (176, 76.5%) stated they were willing to shower or bathe within two hours of outdoor activities. And most (153, 66.5%) were willing to apply treatments to their pets to prevent tick bites (153, 66.5%). Drying clothing or outdoor gear in a dryer on high heat (82, 35.7%) and wearing clothing pretreated with a bug repellent, such as permethrin (50, 21.7%) were less accepted. About one third of participants were willing to use either natural or synthetic repellents (72, 31.3%). Natural repellents were more widely accepted (124, 53.9%) than synthetic repellents (108, 47.0%). About one third of the sample was unwilling to use either kind of repellent (69, 30.0%)

### 3.5. Outreach Impacts and Preferences

Only about a quarter of participants (63, 27.4%) reported awareness of Ohio Department of Health messaging related to TBDs. Participants who were aware of ODH messages were more likely to see Lyme disease as a serious disease and to believe their community to be at risk of TBDs than people who had not seen the messages (see Table 3). These differences were statistically significant and the effect size for each was small (*d* = 0.36, *d* = 0.34, respectively). Participants who were aware of these messages were more likely to believe Lyme disease was serious and to have applied pesticide around their homes (See Table 4). This difference was statistically significant and the effect size was small (*d* = 0.17). They were not more likely to have checked themselves for tick, to have used repellents or to have used devices that apply pesticides to rodents at their home. Most communication channels were ranked somewhere between slightly and moderately helpful for preventing diseases spread by ticks (M_web_ = 3.01, s.d. = 1.07; M_print_ = 2.70, s.d. = 1.09; M_communityresources_ = 2.90, s.d. = 1.07; M_presentations_ = 2.65, s.d. = 1.13; M_email/texts_ = 2.56, s.d. = 1.13; M_apps_ = 2.44, s.d. = 1.22).

## 4. Discussion

This study provides a baseline understanding of KAPs related to TBDs among residents of Athens County, Ohio, a low-incidence area bordering a high-incidence region. The findings highlight gaps in knowledge, inconsistences in risk perception, and limited adoption of tick-bite prevention behaviors, which are critical for shaping effective public health communication interventions.

The participants demonstrated high awareness of Lyme disease but limited familiarity with other TBDs. This finding aligns with previous research indicating that public awareness of non-Lyme borne diseases remains low [2,16]. The perception of tick encounters was generally low, even among those who spent significant time in environments where ticks are present. Despite the increasing prevalence of ticks in Ohio due to climate change [6], most participants did not perceive their household or community to be at significant risk. These findings suggest a disconnect between the scientific evidence of expanding tick habitats and public risk perception. Additionally, studies among professional populations, such as physicians and health department employees, have found that even those in health-related fields often have limited knowledge and do not always adopt prevention behaviors [18,23]. This suggests that public health communication must target not only the general public but also professionals who serve as key sources of information. If the fundamental assumption undergirding KAPs is true, that people must have knowledge of a health threat to have attitudes supportive of preventive action, then the inadequate knowledge and low perception of threat present clear content for messages: educating the public about TBDs and raising their perceptions of threat.

The results also indicate suboptimal engagement in tick-bite prevention behaviors. While a substantial proportion of participants reported performing tick checks and using repellents, very few did so consistently. The most commonly reported barrier was forgetting to engage in preventative behaviors, rather than concerns about cost or safety. This suggests that increased reminders, rather than simply increasing awareness, may be necessary to promote sustained adoption of protective measures. Additionally, engagement in environmental prevention, such as applying pesticides around the home, was low, largely due to concerns about cost, safety, and awareness of available options. These findings are consistent with prior studies demonstrating that knowledge alone does not drive behavior change; attitudes and perceived barriers significantly influence preventive practices [1,3]. These results are also similar to national findings in other low-incidence states that border high incidence states [41] where the adoption of tick-bite prevention methods was suboptimal. Additionally, similar to previous research among adults in low-Lyme incidence subregions, such as the St. Louis metropolitan area [1], southern Illinois farming communities [39], and Indiana [40], that are near Lyme-endemic areas in northern Illinois and Michigan, we found low-to-moderate levels of knowledge, attitudes unsupportive of preventive action, and under-adoption of preventive action. Our findings differ from Omodior and colleagues’ findings of significant uptake of preventive behaviors among parents in Indiana [26]; that study, however, was for other protective behaviors of children and, as they acknowledge, subject to multiple forms of social desirability bias. Similar to these previous studies, we believe addressing risk perceptions that could become supportive of preventive action is therefore necessary in future interventions.

Only about a quarter of participants reported awareness of Ohio Department of Health messaging related to TBDs. Those who were aware were more likely to perceive Lyme disease as serious and their community as at risk. However, this awareness did not significantly correlate with self-checks or repellent use, suggesting that current messaging strategies may not be effectively promoting behavior change. Indeed, current public messaging by the Ohio Department of Natural Resources [42] and the Ohio Department of Public Health [45] emphasizes identification of ticks and preventive actions that could be taken, but does not emphasize *why* a person would want to avoid being bitten by a tick. Because KAP models argue that there needs to be both knowledge and attitudes supportive of behavioral change to see a change in practice, public health agencies in Ohio may wish to be more explicit about the relationships among tick presence, tick bites, and disease causality to encourage changes in practice. In addition, our results suggest that current messages do not reach their audience. Given the preference for digital communication methods indicated in this study, incorporating tailored web-based, text, and app-based reminders may enhance engagement with tick prevention recommendations. Public health agencies in Ohio currently rely on traditional media (print, billboards, radio, and television) or on “pull” messaging on new media where the user seeks out the information. The audience preferences expressed in our study, however, indicate that “push” media, where the message sender sends electronic communication to users who have not explicitly requested it, may increase reach of the message. Text and embedded app-based messages can be sent by governmental agencies with little friction and may be a way to increase the reach of messages into non-endemic regions where active information-seeking by residents is unlikely. Research in Lyme-endemic areas has demonstrated that targeted educational interventions can significantly improve adherence to tick-bite prevention practices [17], indicating that refining outreach strategies in low-incidence areas could be similarly beneficial.

The findings underscore the need for targeted health communication strategies in low-incidence regions to proactively address emerging risks. Given the expansion of tick populations into Ohio [42], early intervention efforts in naïve populations like those in Athens County may be critical in preventing the establishment of endemic tick-borne disease cycles. Prior studies emphasize the need for proactive messaging in low-incidence states bordering high-incidence areas [44], yet southeast Ohio remains largely overlooked in this regard. Future public health initiatives should prioritize increasing awareness of non-Lyme TBDs, addressing behavioral barriers such as forgetfulness through environmental cues and reminders, and tailoring messaging to the preferred communication channels of at-risk populations. Additionally, community-based interventions that emphasize practical, low-effort preventive strategies may improve adherence to tick-bite prevention behaviors. This study highlights some strategies that may be effective in Athens County, Ohio and transferable to other similar regions in broader tick prevention efforts.

Despite clear lessons from KAPs in a low-Lyme-endemic region, this study was conducted in a single county, which may limit the generalizability to other low-incidence areas. The use of a cross-sectional, non-probability sample design also introduces potential biases in representation and accuracy. In particular, the sample for this study was generally young and generally well-educated, and applicability to older and less educated populations may be limited. The behavioral items were all self-report items, introducing potential social desirability or recall biases into the data. Larger samples may also be intentionally designed for broader demographic representation and allow for the use of multivariate analysis to control for potentially confounding demographic variables. Although our questionnaire and analytic design choices were modeled after previous research [2], adding observational variables and multivariate techniques may enhance the findings. In addition, to account for the limitations of cross-sectional design, future research should expand to other regions in the lower Midwest and employ longitudinal designs to assess changes in KAPs over time. Additionally, experimental studies testing the efficacy of different messaging strategies in promoting behavior change would be beneficial. Given the increasing spread of ticks due to climate change [7], further studies should also explore how climate perceptions influence public engagement with tick prevention strategies.

## 5. Conclusions

As tick populations expand and TBDs pose increasing risks in historically low-incidence areas, understanding and addressing public perceptions and behaviors become critical. This study provides foundational insights for designing effective public health interventions tailored to naïve populations. By enhancing awareness, addressing behavioral barriers, and optimizing communication strategies, public health efforts can improve tick-bite prevention practices and mitigate the risks associated with TBDs in Ohio and similar regions. Expanding research efforts beyond endemic areas will ensure that preventive interventions are in place before disease incidence rises, rather than in response to an established public health crisis.

## Figures and Tables

**Table 1:** Demographics.

Characteristic	N	%
Type of Residence		
Urban	20	8.7
Suburban	64	27.8
Town	116	50.4
Rural	30	13.0
Gender		
Female	142	61.7
Male	85	37.0
Other	3	1.3
Ethnicity		
Hispanic	8	3.5
Non-Hispanic	215	93.4
Race		
American Indian or Alaska Native	1	0.4
Asian	2	0.9
Black or African American	11	4.8
Native Hawaiian or Other Pacific Islander	2	0.9
White	215	93.5
Level of Education		
Never attended	0	
Some high school or less	1	0.4
Complete high school/GED	28	12.2
Trade school	1	0.4
Some college	157	68.3
Associate’s or 2-year degree	6	2.6
Bachelor’s or 4-year degree	26	11.3
Master’s degree	7	3.0
Advanced degree such as PhD, law degree or medical degree	2	0.9
Income		
<25,000	107	46.5
$25,001–40,000	8	3.5
$40,001–55,000	5	2.2
$55,001–65,000	5	2.2
$65,001–75,000	8	3.5
$75,001–100,000	17	7.4
>$100,000	32	13.9
Age		
Range = 18–81	M = 22.3 (s.d. = 7.5)	
18–29	219	95.2%
30–39	5	2.2%
40–49	1	0.4%
50–59	4	1.7%
60+	3	1.3%

Note: As participants could opt not to answer demographic questions, and could select more than one race, totals may not add to 230.

**Table 2:** Awareness of and perceived seriousness of selected tick-borne disease.

Disease	Reporting No Awareness N (%)	Reporting Awareness N (%)	Perceived Seriousness M (s.d.)
Lyme disease	10 (4.3%)	220 (95.7)	4.05 (1.16)
Anaplasmosis	207 (90.0%)	23 (10.0%)	1.36 (1.91)
Babesiosis	214 (93.0%)	16 (7.0%)	1.28 (1.87)
Ehrlichiosis	216 (93.9%)	13 (5.7%)	1.22 (1.84)
Powassan virus disease	217 (94.3%)	12 (5.2%)	1.30 (1.90)
Rocky Mountain spotted fever	147 (63.9%)	82 (35.7%)	1.81 (2.04)

**Table 3:** Means and *t*-tests among -related tick attitudes and reported adoption of preventive practice.

	Exposure M (s.d.)	Seriousness of Lyme Disease M (s.d.)	Community Risk of Disease M (s.d.)	Household Risk of Disease M (s.d.)
Checks Self ^†^	***p* < 0.01,** **d = 0.60**	*p* = 0.26, d = −0.16	***p* < 0.01,** **d = −0.55**	***p* < 0.01,** **d = −0.47**
Always/Most of the Time (n = 72)	2.38 (0.99)	4.18 (1.18)	2.83 (1.06)	1.93 (0.86)
Less Often (n = 154)	3.01 (1.08)	3.99 (1.15)	2.30 (0.94)	1.56 (0.75)
Uses Repellent ^†^	*p* = 0.41, d = −0.12	*p* = 0.56, d = 0.09	***p* < 0.01,** **d = 0.48**	*p* = 0.44, d = 0.14
Always/Most of the Time (n = 64)	2.72 (1.15)	4.13 (1.19)	2.81 (0.92)	1.75 (0.78)
Less Often (n = 161)	2.85 (1.06)	4.02 (1.16)	2.34 (1.01)	1.66 (0.82)
Treated Home with Pesticide	*p* = 0.35, d = 0.17	*p* = 0.91, d = 0.02	***p* < 0.01,** **d = −0.58**	***p* < 0.01,** **d = −0.64**
Yes (n = 36)	2.69 (1.26)	4.03 (1.16)	2.97 (0.94)	2.11 (0.98)
No (n = 194)	2.89 (1.14)	4.05 (1.16)	2.40 (1.00)	1.61 (0.74)
Treated Home with Device	*p* = 0.51, d = −0.22	*p* = 0.31, d = 0.34	*p* = 0.06, d = −0.65	***p* < 0.01,** **d = −1.00**
Yes (n = 9)	3.11 (1.62)	3.67 (1.73)	3.11 (1.17)	2.44 (0.73)
No (n = 221)	2.85 (1.14)	4.06 (1.12)	2.46 (1.00)	1.66 (0.79)
Aware of ODH Messages	*p* = 0.43, d = 0.12	***p* = 0.02,** **d = −0.36**	***p* = 0.03,** **d = −0.34**	*p* = 0.90, d = −0.02
Yes	2.76 (1.04)	4.35 (1.10)	2.73 (0.85)	1.70 (0.82)
No	2.90 (1.20)	3.93 (1.16)	2.40 (1.05)	1.68 (0.80)

NOTE: ^†^ only among persons reporting time spent in areas with ticks, significant differences marked in **bold**.

**Table 4:** Chi-Square analysis of association among campaign exposure and reported preventive practices.

	Checks Self Always/Most ^†^	Less Often ^†^	Uses Repellent Always/Most ^†^	Less Often ^†^	Treat Home with Pesticide	No	Treat Home with Device	No
Campaign Exposure	39	115	42	119	20	147	5	58
No Campaign Exposure	24	48	21	43	16	47	4	163
x-square value (d.f.)	1.57 (1, 226) *p* = 0.21, v = 0.08		1.03 (1, 225) *p* = 0.31, v = 0.07		**6.24 (1, 230)** ***p* = 0.01,** **v = 0.17**		3.74 (1, 230) *p* = 0.05, v = 0.13	

NOTE: ^†^ only among persons reporting time spent in areas with ticks, significant associations marked in **bold**.

## Data Availability

The original data presented in the study are openly available in OSF at https://osf.io/hjqz4/files/osfstorage/6824ba0611359804f07a1b96.
